# Molecular characterization and epidemiology of *Streptococcus pneumoniae* serotype 24F in Denmark

**DOI:** 10.1038/s41598-019-41983-8

**Published:** 2019-04-02

**Authors:** Ioanna Drakaki Kavalari, Kurt Fuursted, Karen A. Krogfelt, H.-C. Slotved

**Affiliations:** Department of Bacteria, Parasites and Fungi, Statens Serum Institut, Copenhagen, Denmark

## Abstract

Since 2012, have we in Denmark observed an increase of invasive pneumococcal infections (IPD) due to *Streptococcus pneumoniae* serotype 24F. We here present epidemiological data on 24F IPD cases, and characterization of 48 24F clinical isolates based on clonal relationship, antimicrobial resistance (AMR) determinants and virulence factors. IPD surveillance data from (1999–2016) were used to calculate the incidence and age-distribution of serotype 24F IPD and the effect of pneumococcal conjugated vaccines (PCV). Characterization of forty-eight 24F isolates (14.7% of all 24F isolates from the period) was based on whole-genome sequencing analysis (WGS). The IPD cases of serotype 24F showed a significant increase (p < 0.05) for all age groups after the PCV-13 introduction in 2010. The majority of tested 24F isolates consisted of two MLST types, i.e. the ST72 and the ST162. Serotype 24F IPD increased in Denmark after the PCV-13 introduction in parallel with an increase of the ST162 clone. The genotypic penicillin binding protein (PBP) profile agreed with the phenotypical penicillin susceptibility. The virulence genes *lytA*, *ply*, *piaA*, *piaB*, *piaC*, *rspB* and the *cpsA*/*wzg* were detected in all 24F isolates, while the *pspA* and *zmpC* genes were absent.

## Introduction

*Streptococcus pneumoniae* is a ubiquitous bacterium present in the commensal bacterial community in the human nasopharynx. It is responsible for non-invasive infections as well as invasive pneumococcal disease (IPD) with high morbidity and mortality especially among young children and the elderly^1^. The introduction of the pneumococcal conjugated vaccine (PCV) provided an effective protection against IPD in children. The first PCV on the global market was Prevenar 7 (PCV-7) (Pfizer Vaccines) in 2000 including seven different serotypes (4, 6B, 9V, 14, 18C, 19F and 23F), followed by the 10-valent pneumococcal-conjugate vaccine (PCV-10) (Synflorix, GlaxoSmithKline Biologicals) in 2009 including the serotypes 1, 4, 5, 6B, 7F, 9V, 14, 18C, 19F and 23F, and by the Prevenar 13 vaccine (PCV-13) (Pfizer Vaccines) in 2010 including the serotypes 1, 3, 4, 5, 6A, 6B, 7F, 9V, 14, 18C, 19A, 19F and 23F^1^. Besides the conjugate vaccines, a pneumococcal polysaccharide vaccine (PPV-23) (Pneumovax®, Merck) based on purified capsular polysaccharides from 23 different serotypes, was introduced in 1996. The PPV-23 vaccine is recommended for patients older than 2 years of age and of high risk for IPD and for the 65+ years age group^2,3^.

With the introduction of PCV, a significant reduction of IPD cases caused by the included PCV serotypes was seen, but at the same time serotype replacement was observed, with the appearance of new pneumococcal serotypes not included in the vaccines^1^. One of the non-PCV serotypes that has emerged in recent years is serotype 24F^4,5^. The non-vaccine serotype 24F is responsible for many penicillin non-susceptible related IPD cases^6^. Serotype 24F is also linked to erythromycin-clindamycin and tetracycline resistance and is one of the main serotypes that was recovered from young children with IPD between 2011 and 2012 in France^7^. After the introduction of PCV13 in 2010 in France, serotype 24F, including other non-vaccine serotypes (10A, 12F and 15A), were responsible for approximately 39% of pneumococcal meningitis (PM) cases in children below 5 years of age from 2012 to 2014^8^.

During the last five years, a small increase of IPD cases with serotype 24F has been observed in Denmark^1^. Because the serotype 24F is an emerging non-pneumococcal vaccine serotype, which to our knowledge is not a part of any pneumococcal vaccine or as part of planned future pneumococcal vaccine. Is it therefore essential to monitor the epidemiology and susceptibility of serotype 24F, and to provide information to the international community that measures against serotype 24F are needed.

The intention of this study was to present epidemiological data based on IPD cases in Denmark in the period from 1999 to 2016. Furthermore, to present a detailed characterization of forty-eight 24F isolates (representing 14.7% of all 24F isolates from the period) using whole-genome sequencing analysis (WGS). Thus, the serotype 24F’s clonal relationship over the years and antimicrobial resistance determinants are presented. Finally, the presence of capsular, toxin and surface related genes are suggested as pneumococcal species.

## Material and Methods

### Strain collection

**Figure 1:**
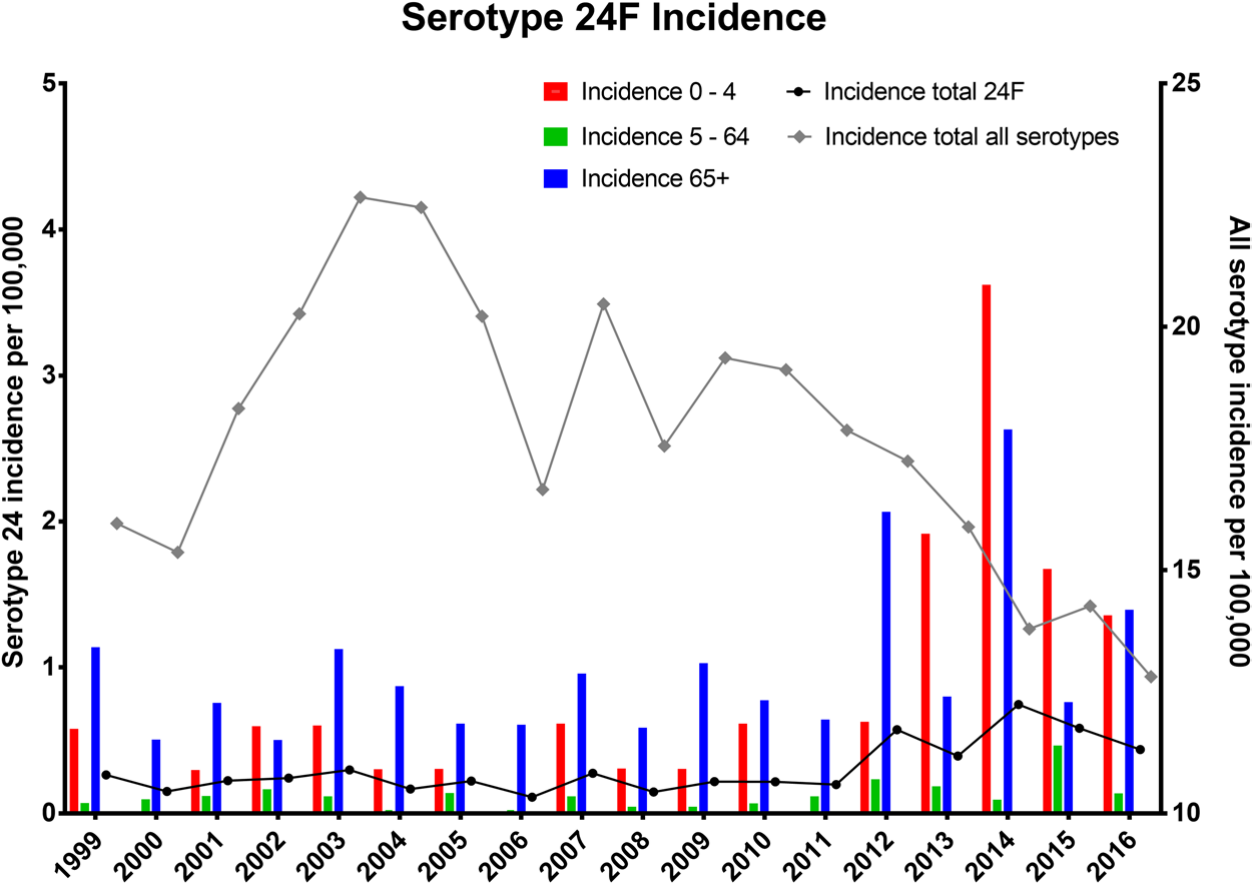
The IPD incidence in Denmark from 1999 to 2016. The IPD incidence for serotype 24F in all three age groups from 1999 until 2016. Red bars (left Y-axe) represent 0–4 years, green bars (left Y-axe) represent 5–64 years and blue bars (left Y-axe) represent 65+ years. The black curve (left Y-axe) represents the total serotype 24F incidences per year. The gray curve (right Y-axe) represent the total IPD incidences for all serotypes per year.

All invasive pneumococcal isolates of serotype 24F from 1999 to 2016 were retrieved from the Danish laboratory surveillance system at the national Neisseria and Streptococcus Reference Laboratory (NSR), Statens Serum Institut (SSI) (Supplementary Table 1). All the IPD serotype 24F cases reported were isolated form patients diagnosed with either bacteraemia (from blood) or meningitis (from cerebrospinal fluid). An IPD case was defined as the occurrence of *S. pneumoniae* in cerebrospinal fluid, blood or other normally sterile sites^9^. Incidence and Incidence Rate Ratio (IRR) data from 24F from 1999 to 2014 was previously presented in Slotved *et al*.^1^ including information on age, sex, serotype and origin of the pneumococcal isolate (blood, cerebrospinal fluid etc.). Data on the total IPD cases for all age groups in Denmark are presented in Fig. 1 to compare with the serotype 24F. Detailed data on the total IPD cases for all age has previously been presented^1,10^.

### Identification of pneumococcal isolates

The pneumococci isolates were phenotypically identified by optochin susceptibility and bile solubility tests^1,11^. All isolates were serotyped either by the Quellung reaction alone or by the Pneumotest Latex kit (SSI-Diagnostica, Copenhagen, Denmark) combined with the Quellung reaction using type-specific pneumococcal rabbit-antisera as previously described (SSIDiagnostica, Copenhagen, Denmark)^11^.

### Characterization of 48 clinical selected isolates

Forty-eight *Streptococcus pneumoniae* 24F isolates were selected for detailed characterization using whole genome sequencing (Table 1). Forty-seven 24F isolates were isolated from blood and spinal fluid in the period 1999 to 2017, and one historical isolate 24F was cultured from the trachea of an eight-year-old boy in 1943. The 47 isolates were selected to represent 26 children below five years of age and 21 elderly persons above 64 years of age. Otherwise the isolates were randomly selected during the period 1999–2017 to provide a molecular picture of the serotype 24F isolates in Denmark. The isolates represent 14.7% of the total number of 24F IPD cases during  the period.

**Table 1:** Characteristics of the 47 invasive 24F isolates and one trachea 24F historical isolate from 1943.

Year	Strain number	Age	Sex	Sample type	Capsular genes (Genotype)	MLST (ST)	PEN* (mg/L)	ERY* (mg/L)	CLI* (mg/L)	*ermB*^d^ (Tn family)	*tetM*^d^ (Tn family)	PBP 1a	PBP 2b	PBP 2x
1943	24F-1943	8	Male	Trachea	24F	7179	S (0.032)	S (28.22)	S (25.06)	NR	NR	0	0	0
1999	0630-1999	0	Female	Spinal fluid	24 Group	72	S	S	S	NR	NR	2	0	0
2002	1030-2002	1	Male	Blood	24F	72	S	S	S	NR	NR	2	0	0
2003	0373-2003	0	Female	Blood	24 Group	(12-19-2-?-6-22-14)^b^	R (1.0)	R (7.42)	R (6.77)	+(none)^c^	+(Tn1545)	17	15	22
2004	1186-2004	0	Female	Blood	24F	72	S (0.047)	S (27.99)	S (24.08)	NR	NR	2	0	0
2004	1073-2004	75	Female	Blood	24F	72	S	S	S	NR	NR	2	0	0
2005	0527-2005	0	Male	Spinal fluid	24F	72	S	S	S	NR	NR	2	0	0
2005	0741-2005	78	Male	Blood	24 Group	72	S	S	S	NR	NR	2	0	0
2006	0194-2006	86	Female	Blood	24 Group	72	S (0.032)	S (27.45)	S (24.47)	NR	NR	2	0	0
2006	0579-2006	79	Male	Blood	24 Group	72	S	S	S	NR	NR	2	0	0
2006	1119-2006	75	Male	Blood	24F	72	S	S	S	NR	NR	2	0	0
2007	0834-2007	4	Female	Blood	24F	162	S (0.047)	S (29.11)	S (24.59)	+(none)^c^	+(Tn916)	2	0	28
2007	0258-2007	92	Female	Blood	24 Group	72	S	S	S	NR	NR	2	0	0
2007	1134-2007	83	Female	Blood	24 Group	72	S	S	S	NR	NR	2	0	0
2008	0266-2008	1	Female	Spinal fluid	24F	11100	S	S	S	NR	NR	2	18	0
2008	1108-2008	86	Female	Blood	24 Group	230	I (0.75)^a^	R	R	+(none)^c^	+(Tn1545)	17	15	22
2009	0811-2009	1	Male	Blood	24F	162	S (0.032)	S (0.032)	S (25.31)	+(none)^c^	+(Tn916)	2	0	1
2009	0273-2009	86	Female	Blood	24 Group	72	S	S	S	NR	NR	2	0	0
2009	1199-2009	94	Male	Blood	24 Group	72	S	S	S	NR	NR	2	0	0
2010	0805-2010	1	Male	Spinal fluid	24F	72	S	S	S	NR	NR	2	0	0
2010	0510-2010	2	Male	Blood	24 Group	72	S	S	S	NR	NR	2	0	0
2010	0993-2010	88	Female	Blood	24 Group	72	S	S	S	NR	NR	2	0	0
2011	1853-2011	91	Male	Blood	24F	11131	S	S	S	NR	NR	0	0	0
2012	0263-2012	1	Male	Blood	24F	162	S	S	S	NR	NR	2	0	0
2012	0272-2012	76	Female	Blood	24F	72	S	S	S	NR	NR	2	0	0
2012	0390-2012	72	Male	Blood	24 Group	72	S	S	S	NR	NR	2	0	0
2013	0682-2013	1	Male	Blood	24F	11100	S	S	S	NR	NR	2	18	0
2013	0699-2013	1	Female	Blood	24F	4253	I (0.5)	R (0.0)	R (0.1)	+(none)^c^	+(Tn1545)	17	15	117
2013	0128-2013	75	Female	Blood	24 Group	72	S (0.032)	S (29.31)	S (24.25)	NR	NR	2	0	0
2013	0366-2013	76	Male	Blood	24 Group	72	S	S	S	NR	NR	2	0	0
2014	0149-2014	1	Male	Spinal	24F	162	S	S	S	NR	NR	2	0	0
2014	0404-2014	1	Male	Blood	24 Group	162	S (0.03)	R (0.1)	R (0.1)	+(none)^c^	+(Tn916)	2	0	28
2014	0669-2014	1	Female	Spinal fluid	24F	11100	S	S	S	NR	NR	2	18	0
2014	0747-2014	0	Female	Blood	24 Group	162	S (0.03)	R (0.1)	R (0.1)	+(none)^c^	+(Tn916)	2	0	28
2014	0100-2014	87	Female	Blood	24 Group	72	S	S	S	NR	NR	2	0	0
2015	0090-2015	1	Female	Blood	24 Group	72	S (0.016)^a^	S	S	NR	NR	2	0	0
2015	0096-2015	1	Male	Blood	24F	162	S (0.032)^a^	S	S	NR	NR	2	0	0
2015	0182-2015	1	Female	Blood	24 Group	11100	S (0.016)^a^	S	S	NR	NR	2	18	0
2015	0233-2015	1	Male	Spinal fluid	24 Group	4253	I (0.5)	R (0.1)	R (0.1)	+(none)^c^	+(Tn1545)	17	15	116
2015	0641-2015	1	Male	Spinal fluid	24 Group	162	S (0.016)^a^	S	S	NR	NR	2	0	0
2015	0120-2015	70	Male	Blood	24F	162	S (0.016)^a^	S	S	NR	NR	2	0	0
2015	0223-2015	81	Female	Blood	24F	162	S (0.03)	R (0.1)	R (0.1)	+(none)^c^	+(Tn916)	2	0	28
2016	0104-2016	4	Female	Blood	24F	72	S	S	S	NR	NR	2	0	0
2016	0201-2016	2	Male	Blood	24 Group	162	S	S	S	NR	NR	2	0	0
2016	0041-2016	71	Female	Blood	24 Group	162	S	S	S	NR	NR	2	0	0
2016	0350-2016	68	Male	Blood	24 Group	162	S (0.03)	R (0.2)	R (0.2)	+(none)^c^	+(Tn916)	2	0	28
2017	0068-2017	1	Female	Blood	24F	(7-11-10-1-6-?-14)^b^	S (0.03)	R (0.1)	R (0.2)	+(none)^c^	+(Tn916)	2	0	28
2017	0345-2017	1	Male	Blood	24 Group	162	S (0.03)	R (0.1)	R (0.1)	+(none)^c^	+(Tn916)	2	0	28

^*^Isolates tagged with and S (sensitive) or R (resistance) was only tested with disk diffusion. For penicillin was S/R tagged only based on oxacillin zone diameter, penicillin test was not performed on the isolates. For isolates with an MIC value was the sensititre test performed, except for a few isolates tagged with an “a”.

^a^Penicillin Etest.

^b^Has been submitted to the curators of the MLST database.

^c^Did not harbor any mobile elements of the Tn-family^37^.

^d^NR stands for “not relevant” and tag the isolates where *ermB* and *tetM* genes were not detected and Tn linkage therefore is not relevant.

### Molecular species identification

The isolates were sequenced by paired-end Illumina sequencing. Genomic DNA was extracted using a DNeasy Blood & Tissue Kit (QIAGEN, Hilden, Germany) and fragment libraries were constructed using a Nextera XT Kit (Illumina, Little Chesterford, UK) followed by 250-bp paired-end sequencing (MiSeqTM; Illumina) according to the manufacturer’s instructions. The paired-end Illumina data were de novo assembled using CLCbio’s Genomics Workbench v.7.5 QIAGEN) reporting only contigs >500 bp using standard settings.

Bioinformatics, including Blast was done using the software the CLC Main Workbench (Version 7.9.1, www.qiagenbioinformatics.com).

All 48 isolates were species identified according to the description by Scholz *et al*.^12^. Briefly, the 16S rRNA sequence (accession number: AY485600) identified by Arbique *et al*.^13^ was used to identify the nucleotide position at 203^12^. The *S. pneumoniae* identification was based on the location of cytosine at the 203 position, while the existence of adenine-residues suggested that the species belonged to another *Streptococcus* species^12^.

The presence/absence of a gene was based on a cut-off of 80% coverage and a 95% identity for a positive gene detection in this study^14^.

The genomic sequence data for the 48 isolates are deposited in the Genbank (https://www.ebi.ac.uk/ena) (ENA project no. PRJEB31691).

The species identification was confirmed by Multilocus Sequence Analysis (MLSA) analysis using the 9 housekeeping genes (*ddl*, *gdh*, *rpoB*, *sodA*, *map*, *pfl*, *ppaC*, *pyk*, *tuf*) as described by Bishop *et al*.^15^ and Killian *et al*.^16^.

### Molecular characterization of their capsular genes

Sequences from all 48 isolates were checked for the 92 capsular polysaccharide genes (CPS genes) by BLAST. The FASTA files for the capsular locus sequences were retrieved from the NCBI database, using the accession numbers CR931632-CR931722, JF911515.1 and HV580364.1^17,18^.

Identification of serogroup/type 24F was performed according to the presence/absence of genes using a cut-off of 80% coverage and a 95% identity as described by Sheppard *et al*.^14^ and Kapatai *et al*.^18^. However, differentiating further into a specific serotype within the group 24 can be difficult^18^. We therefore only presented genotypes, where the coverage and identity clearly indicated a specific genotype.

### Multilocus sequence typing (MLST)

MLST was performed using the PubMLST DataBase (https://pubmlst.org/spneumoniae/) to identify the sequence type (ST) for each of the 24F *S. pneumoniae* strains. Analysis of the STs and assignment to CC was performed using PHYLOViZ 2.0 programme (http://phyloviz.readthedocs.io/en/latest/#). The STs that shared at least six of seven allelic variants composed a CC (clonal complex)^19^.

A phylogenetic tree based on single nucleotide polymorphisms (SNP’s) analysis of the core genome was performed on the 48 isolates. Identification of SNP’s’ was performed using BWA-mem for mapping and GATK with filtering set to remove positions with less than 10-fold depth and 90% unambiguous variant calls as implemented in NASP^20^ against isolate 0100-2014’s chromosome, which was used as a reference strain in the SNP alignment after removal of duplicated regions using NUCmer. The resulting SNP matrix was purged for recombination using Gubbins^21^. FigTree v1.4.3 (http://tree.bio.ed.ac.uk/software/figtree/, accessed 25-09-2018) was used to visualize the phylogenetic tree.

### Antibiotic susceptibility testing (phenotypic tests)

Screening of antibiotic susceptibility was performed on all 48 isolates by disk diffusion using Mueller–Hinton 5% blood agar with NAD (Oxoid, Denmark) incubated in ambient air with 5% CO_2_ at 35 °C and oxacillin, penicillin, erythromycin and clindamycin discs (Oxoid, Denmark). Some isolates were also tested using the E-test randomly, however not on a regularly basis. Isolates showing non-susceptibility were tested using a Microbroth dilution test (Sensititre, Streptococcus species MIC Plate, STP6F, Trek Diagnostic System, USA).

MIC determination of penicillin G was done by using either a gradient test (Etest; bioMérieux), before 2010, or a broth microdilution method (Sensititre, Streptococcus species MIC Plate, STP6F, Trek Diagnostic System, USA), after 2010. *S. pneumoniae* ATCC 49619 was used as a quality control strain. Interpretation of susceptibility was done according to the breakpoints described in EUCAST (http://www.eucast.org/clinical_breakpoints/).

We do not routinely perform phenotypical screening of tetracycline on the isolates.

### Genotypic antibiotic resistance profile

Penicillin (PEN) susceptibility in pneumococci is associated with penicillin-binding proteins (PBP) which in penicillin non-susceptible strains of pneumococci are modified to low-binding-affinity versions of the native PBP1A, PBP2B and PBP2X. The 48 isolates were analyzed for their PBP signature, based on a genotyping proposal and algorithm described for PBP1A, PBP2B and PBP2X^22^, where the combination of the three PBP signatures determines the level of beta-lactam resistance. The 48 isolates were tested by BLAST with the published types of predictive mutations vs. resistance levels of PBP1A, PBP2B and PBP2X proteins as described in Li *et al*.^22^ (Table 1).

### Erythromycin (ERY), clindamycin (CLI) and tetracycline (TET) genotypic profile

The 48 *S. pneumoniae* genomes were analyzed for the genes *ermB* (NCBI, FJ667782)*, mefA* (NCBI, KU739790)*, mefE* (NCBI, NC_003098.1 (R6), and Tn916 gene *tet*(M) (FR671418)^23^. ResFinder 2.1 (https://cge.cbs.dtu.dk/services/ResFinder/) (80% ID threshold and 60% minimum length settings)^24^ was used to confirm the presence of the three genes.

### Virulence gene profile

Presence of capsular, toxin and surface related genes used for pneumococcal virulence characterization^25^ and presence of genes with potential species discrimination:

The presence of the virulence genes *lytA* and *ply*^26^ for the identification of *S. pneumoniae* were tested as recommended by Centers for Disease Control (CDC)^12,27^.

Genes *piaA*, *piaB* and *piaC* (GenBank: AF338658.1) coding for membrane proteins and ATP-binding proteins as previously described^14,27^.

Genes *zmpB*^28^ and *zmpC* (AE005672:75858-76420)^29^, which are paralogous zinc metalloproteases^30^.

Genes for the pneumococcal surface proteins (*pspA*) (Genbank: AF516671)^31^ and *psrp*^32^.

The partial capsular gene *cpsA*, also known as the *wzg* gene, was tested for its presence/absence (Genbank: AF057294:2134–2473)^33^.

Gene *rpsB* encoding for ribosomal protein S2^29^.

### Data analysis

Data were analyzed using Graph Pad Prism version 7 (GraphPad Software) for descriptive statistical analysis. RStudio version 1.1.447 and R version 3.5.0 for Windows was used for all calculations of incidence rate, incidence rate ratio (IRR) and confidence interval (CI) (http://www.r-project.org/). Two tailed Fisher’s Exact Test in R was used to calculate P-values. P < 0.05 was considered significant. The raw data for calculation of the incidence rate are presented in the Supplementary Table 1.

### Ethical considerations

The study was a retrospective, population-based study based on national laboratory surveillance data on isolates from patients with IPD. Since data and samples from patients were collected routinely for national surveillance purposes, no ethical approval or informed consent from patients or guardians were required. The study was approved by the Danish Data Protection Agency (record number 2007-41-0229).

## Results

### Prevalence/incidence of invasive pneumococcal disease in 1999–2016 due to *S. pneumoniae* serotype 24F

Data from the incidence rates of serotype 24F IPD cases from 1999 until 2016 are presented in Fig. 1 and Table 2. The incidence of serotype 24F IPD cases was low in the period from 1999–2007 (0.22 per 100.000, CI: 0.17–0.26) and 2008–2010 (0.19 per 100.000, CI: 0.09–0.30), while an increase in incidence (0.49 per 100.000, CI: 0.29–0.69) was observed in all age groups from 2011 to 2016. The introduction of PCV-7 in 2007 did not affect the 24F incidence in any of the age groups, while the introduction of PCV-13 in 2010 showed a significant increase in prevalence, two years after the introduction. The increase in the IRR of serotype 24F IPD varied with an IRR of 3.69 (CI: 1.30-10.53) in infants (P = 0.0083), 3.78 (CI: 1.73-8.32) in the age group from 5 to 64 years ((P < 0.001) and 1.74 (1.74 CI: 1.08–2.80) in the age group +65 (P = 0.025), although the general mean incidence was very low.

**Table 2:** The mean incidence rates and 95% confidence intervals are presented for serotype 24F for all three age groups and in total. Incidence Rate Ratios (IRRs) are calculated by dividing a mean incidence from one period by a mean incidence from another period. IRR values were statistically significant (P < 0.01) calculated using a two tailed Fisher’s Exact Test.

Age	1999–2007 (Before introduction of PCV-7)	2008–2010 (After introduction of PCV-7 and before introduction of PCV-13)	2011–2016 (After introduction of PCV13)	1999–2007 versus 2008–2010	2008–2010 versus 2011–2016
	Mean incidence of serotype 24F per 100,000 95% confidence interval of the mean incidence (lower CI; upper CI)			IRR, 95% confidence interval of the mean incidence (lower CI; upper CI) Two tailed Fisher’s Exact Test	
0–4 years	0.37 (0.17–0.56)	0.41 (−0.03–0.85)	1.53 (0.22–2.84)	1.11 (0.35–3.50) P = 0.77	3.69 (1.30–10.53) P = 0.0083*
5–64 years	0.10 (0.06–0.13)	0.05 (0.02–0.09)	0.21 (0.06–0.35)	0.56 (0.25–1.26) P = 0.17	3.78 (1.72–8.32) P = 0.0002*
65 + years	0.79 (0.60–0.98)	0.80 (0.25–1.35)	1.38 (0.53–2.23)	1.01 (0.61–1.67) P = 1	1.74 (1.08–2.80) P = 0.025*
Total all age group	0.22 (0.17–0.26)	0.19 (0.09–0.30)	0.49 (0.29–0.69)	0.89 (0.60–1.33) P = 0.63	2.53 (1.73–3.69) P < 0.00001*

The general IPD incidence for all age groups and serotypes in Denmark was reduced since the introduction of PCV-7 (Fig. 1).

### Identification of the capsular genes for serotype 24F

While all isolates were confirmed phenotypically to be serotype 24F, it was only possible to correctly identify the genotype to group level for 20/48 serotype 24F isolates. For the 28 remaining isolates the 24F capsular genes were correctly identified (Table 1).

### Phylogenetic analysis by MLST

The ST72 belonging to Clonal complex (CC) 72 prevailed among the others STs and included 23 isolates. This was followed by ST162 belonging to CC156 and consisting of 14 isolates.

The CC230 consisted of our isolates (1 ST230, 2 ST4253 and 1 ST(12-19-2-?-6-22-14). Three different singletons ST7179 (1 isolate), ST11100 (4 isolates) and ST4253 (2 isolates) were detected (Table 3).

**Table 3:** Virulence genes detected in the *S. pneumoniae* isolates. These parameters were used for positive gene detection: *Cut-off of overlap as 80% and 95% identity. LytA*^26^, *ply*^29^, *piaA/piaB/piaC* (AF338658), *rpsB* (MF375925), *cpsA*/*wzg* (AF057294:2134–2473) were detected in all 48 isolates. *PspA* (AF516671) and *zmpC* (AE005672:75858–76420)^29^ were not detected in any of the isolates.

Year	Strain number	MLST (ST)/Clonal Complex	*psrP* ^(30)^	*zmpB* ^(26)^
1943	24F-1943	7179/singleton	+	Negative
1999	0630-1999	72/72	+	Negative
2002	1030-2002	72/72	+	Negative
2003	0373-2003	(12-19-2-?-6-22-14)/230	Negative	Negative
2004	1186-2004	72/72	+	Negative
2004	1073-2004	72/72	+	Negative
2005	0527-2005	72/72	+	Negative
2005	0741-2005	72/72	+	Negative
2006	0194-2006	72/72	+	Negative
2006	0579-2006	72/72	+	Negative
2006	1119-2006	72/72	+	Negative
2007	0834-2007	162/156	Negative	+
2007	0258-2007	72/72	+	Negative
2007	1134-2007	72/72	+	Negative
2008	0266-2008	11100/singleton	Negative	Negative
2008	1108-2008	230/230	Negative	Negative
2009	0811-2009	162/156	Negative	+
2009	0273-2009	72/72	+	Negative
2009	1199-2009	72/72	+	Negative
2010	0805-2010	72/72	+	Negative
2010	0510-2010	72/72	+	Negative
2010	0993-2010	72/72	+	Negative
2011	1853-2011	11131/singleton	+	Negative
2012	0263-2012	162/156	Negative	+
2012	0272-2012	72/72	+	Negative
2012	0390-2012	72/72	+	Negative
2013	0682-2013	11100/singleton	Negative	Negative
2013	0699-2013	4253/230	Negative	Negative
2013	0128-2013	72/72	+	Negative
2013	0366-2013	72/72	+	Negative
2014	0149-2014	162/156	Negative	+
2014	0404-2014	162/156	Negative	+
2014	0669-2014	11100/singleton	Negative	Negative
2014	0747-2014	162/156	Negative	+
2014	0100-2014	72/72	+	Negative
2015	0090-2015	72/72	+	Negative
2015	0096-2015	162/156	Negative	+
2015	0182-2015	11100/singleton	Negative	Negative
2015	0233-2015	4253/230	Negative	+
2015	0641-2015	162/156	Negative	+
2015	0120-2015	162/156	Negative	+
2015	0223-2015	162/156	Negative	Negative
2016	0104-2016	72/72	+	Negative
2016	0201-2016	162/156	Negative	+
2016	0041-2016	162/156	Negative	+
2016	0350-2016	162/156	Negative	+
2017	0068-2017	(7-11-10-1-6-?-14)/156	Negative	+
2017	0345-2017	162/156	Negative	+

**Figure 2:**
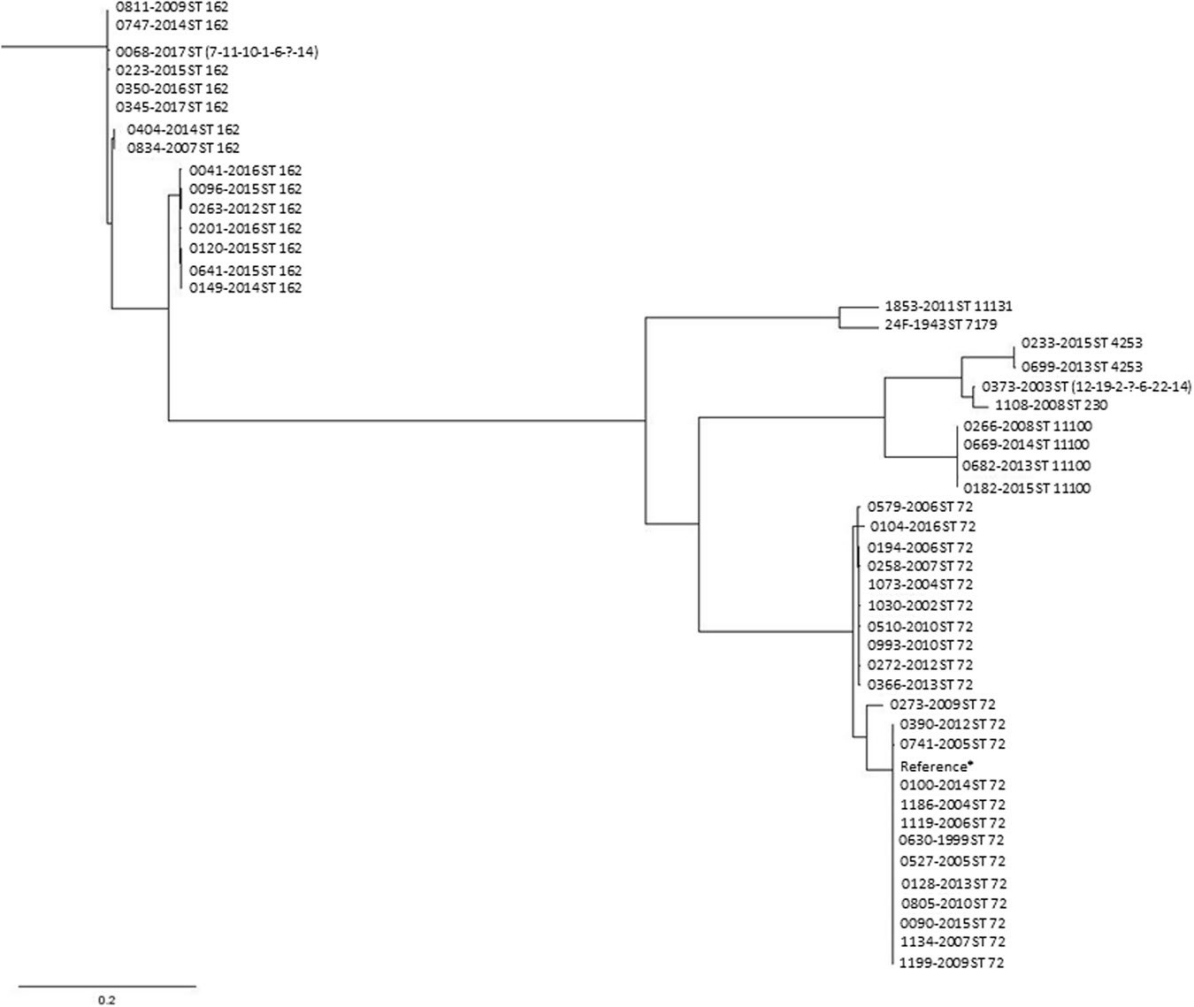
SNP alignment of all 48 isolates based on their SNP site location. *Isolate 0100-2014 was used as a reference strain in the SNP alignment. Strain number and MLST (ST) number have been added to each strain. The observed clusters of isolates contain mainly a cluster of susceptible ST72 and a cluster with cotrimoxazole-resistant ST162 isolates.

MLST sequence types correlated to clade relationships depicted in the core SNP phylogeny (Fig. 2) except for isolate 1186–2004.

### Comparison of phenotypic and genotypic antibiotic resistance profiles

BLAST results of the PBPs protein types and the 48 genomes showed that the majority of the isolates showed combinations of the three PBPs types as previously described^22^, but several new PBP protein type combinations were also observed. The new PBPs combinations were 2-0-28, 2-18-0, 2-0-1, 0-0-0 (all penicillin susceptible) and 17-15-116/117 (both penicillin resistant). Comparison of the PBP combination with the phenotypical penicillin susceptibility (Table 1), showed an agreement with the prediction in the MM model^22^. All the penicillin non-susceptible isolates had the 17 (PBP1a) −15 (PBP2b) -X (The PBP2x showed variable numbers) signatures.

For erythromycin-clindamycin, the BLAST results in general agreed with the phenotypic antibiotic susceptibility result. The presence of the *ermB* gene was linked to the resistance of erythromycin and clindamycin. Only two isolates (0834-2007 and 0811-2009) showed different genotypic and phenotypic results. The ResFinder 2.1 search confirmed the above results (Table 1). The twelve isolates harbouring the *ermB* gene also harboured the *tet(M)* gene according to ResFinder 2.1. Based on the information from the resistance gene accession number provided by the ResFinder 2.1, none of the isolates harbouring the *ermB* gene were found positive for the presence of mobile elements of Tn-family, while both Tn917 (8 isolates) and Tn1545 (4 isolates) was found in the isolates harbouring the *tet(M)* gene (Table 1).

### Characterization of pneumococcal virulence genes

The virulence genes *lytA* and *ply*, the combined genes *piaA*, *piaB*, and *piaC* for membrane and ATP-binding proteins, the *rpsB* gene encoding for ribosomal protein S2, and the partial capsular *cpsA* (*wzg*) gene were detected in all 48 isolates.

The zinc metalloprotease related gene *zmpB* was only detected in isolates from 2007 and forwards.

The surface protein related gene *psrp* was most commonly found in isolates before 2012. Regarding the presence of *zmpB* and *psrp*, forty-one isolates harbored only one of the genes while seven isolates did not show any of the genes, and none harbored both genes.

The pneumococcal surface protein A gene (*pspA*) and the zinc metalloprotease *zmpC* gene were absent from all isolates.

## Discussion

With the introduction of PCV-7 in 2007 in Denmark in the children’s vaccination program, a reduction in IPD with PCV-included serotypes was observed. However, as noted in other countries, non-vaccine serotypes have emerged, and it is therefore important to monitor the appearance of replacement serotypes^1,5,7,34^. Serotype 24F has been observed to be one of the emerging non-PCV serotypes^5,7,28^. Although serotype 24F is only among the 20 most common cause of IPD in Denmark, it is one of the serotypes which have increased most after the PCV-13 introduction in Denmark^1^. Serotype 24F is described as a type with a high potential for invasive disease^5^, and it is therefore an important serotype to keep under surveillance. Figure 1 shows that the total incidence of 24F has been relatively steady until 2012, when a significant increase in serotype 24F IPD cases were observed with a peak in 2014 (Table 2 and Fig. 1). In general, the total IPD in Denmark has been reduced since the introduction of the PCV (Fig. 1)^1,10^.

The analyzed MLST data show, that the majority of the isolates belong to ST72 and ST162. According to the MLST database, ST72 is a well-known clone of serotype 24F. The distribution between the previously described susceptible ST72 and resistant ST162^7^ was also observed in this study (Table 1). ST72 belonged to the penicillin susceptible lineages of CC72, while ST162 (belonging to CC156) also penicillin-susceptible was observed to be cotrimoxazole-resistant and found also among 9 V isolates^7^. The CC156 has emerged after the PCV-13 era^7^, which is also in accordance with our observation of the increase in ST162 (Table 1). The ST162/CC156 are furthermore described as the PMEN3 clone (Spain^9V^-156), particular related to the PCV included serotype 9V and serotype 14^35^. It has been suggested that the appearance of PMEN3 clone of serotype 14 might be due to capsular switching^35^, which also might be the situation with the serotype 24F ST162/CC156 isolates observed in this study.

The ST162 lineage is described as a more successful lineage than the CC72 and the multidrug resistant lineage CC230^7^ (Janoir *et al*. 2016).

Four of the 48 isolates were CC230, one ST230, two ST4253 and a new ST profile (isolate 0373-2003). As described by Janoir *et al*.^7^, CC230 is known as a highly resistant clonal complex, which we can confirm in this study (Table 1). The CC230 has previously been described in relation to a Danish penicillin resistant serotype 14, and are referred to as Denmark^14^-32 PMEN clone. It has also been suggested that the CC230 serotype 24F might be due to capsular switching from a serotype 14 (https://www.pneumogen.net/pmen/, accessed 24th September 2018)^36^. Figure 2 shows that the clustering of isolates based on the SNP site location corresponded well with the MLST.

The historical trachea isolate 24F-1943 was found to harbor a new PBP signature (0-0-0)^22^. The isolate did not show any clonal relationship with the other forty-seven 24F isolates from the period 1999–2017, and it only showed some relation to one other single isolate (isolate 1853–2011) based on the SNP site location (Fig. 2). It is well-known that capsular switching occurs regularly among pneumococcal isolates^36^, and it is therefore a possible explanation that the 24F isolates we see today in Denmark are due to capsular switching in other serotypes^7,36^.

In the study by Li *et al*.^22^, penicillin susceptibility could be predicted by the signature of the three penicillin-binding proteins PBP1a, PBP2b, and PBP2x. We also found an excellent correlation between the signature of the three PBPs and the phenotypical penicillin susceptibility. Interestingly, the four penicillin non-susceptible isolates all had the 17-15-x signature, while all susceptible isolates had low-numbered PBP signatures.

Phenotypic resistance to erythromycin/clindamycin was in general agreement with the genotypic resistance and presence of relevant genes (Table 1). The presence of the *ermB* gene was linked to resistance toward erythromycin and clindamycin and not to the *mefA* and *mefE* genes. The 12 isolates which were positive for the *ermB* gene, also harbored the *tetM* gene. Of note, two isolates harboring both the *ermB* gene and the *tetM* gene were erythromycin susceptible, which has been seen by others^37^.

The selection of virulence genes in this study was inspired by other studies^12,14,26–33^. A more comprehensive list of various pneumococcal genes can be found in the study by Gámez *et al*.^25^. The virulence genes (*lytA*, *ply*, the combined genes *piaA*, *piaB* and *piaC*, and the *cpsA* (*wzg*)), were detected in all 48 isolates, thus confirming their common use for identification of *S. pneumoniae* species using molecular tests^14,26,29,38^ (Table 3).

An interesting observation was made regarding the two genes *pdrp* and *zmpB* (Table 3). None of the 48 isolates harbored both the *psrp* gene and the *zmpB* gene; most of the isolates had only one of the genes, while for seven isolates the two genes were absent. Interestingly, *psrp* was detected in the isolates before 2012. The *psrp* gene has been described as a gene found primarily in antibiotic susceptible strains^36^, which we also observed. Only the MLST ST7179 and the well-known susceptible MLST ST72 strain harbored the *psrp* gene (Table 3). Although the zinc metalloprotease related gene *zmpB* is described as being widespread in *S. pneumoniae*^30^, we only detected the gene in Danish isolates from 2007 and onwards. The *zmpB* gene was only detected in the non-susceptible strains, particularly in ST162 (Table 3). We have not been able to find any studies showing that the *zmpB* gene is linked to antibiotic susceptibility. A 24F isolate with the *zmpB* gene was first detected in 2007 and became more common in the 24F isolates with the increase in the 24F incidence in 2013 (Fig. 1).

## Conclusion

We have seen an increase in serotype 24F IPD in Denmark after the introduction of the PCV-13 vaccine in 2010. It was not significantly associated with an increase in antibiotic resistance or virulence determinants, but was observed in parallel with an increase of the ST162 clone.

## Supplementary information

Dataset 1
