# Supplementary material for: Molecular characterization and epidemiology of Streptococcus pneumoniae serotype 24F in Denmark
Source: Sci Rep. 2019 Apr 2;9:5481. doi: 10.1038/s41598-019-41983-8 (PMC6445336; doi:10.1038/s41598-019-41983-8)
Supplement: Supplementary file 1 — Dataset 1 [file 41598_2019_41983_MOESM1_ESM.docx]

Title:

Molecular characterization and epidemiology of *Streptococcus pneumoniae* serotype 24F in Denmark

Ioanna Drakaki Kavalari^1^, Kurt Fuursted^1^, Karen A. Krogfelt^1^, H-C Slotved^1^*

^1^Department of Bacteria, Parasites and Fungi, Statens Serum Institut, Copenhagen, Denmark

Running title: 24F epidemiology in Denmark

Word count of abstract: 196

Word count of manuscript: 3077

***Corresponding author:**

Hans-Christian Slotved

Department of Bacteria, Parasites and Fungi

Statens Serum Institut

Artillerivej 5, DK-2300 Copenhagen, Denmark

Phone: +45 32688422, Fax: +45 32683865, E-mail: hcs@ssi.dk

Supplementary table 1. Raw data for serotype 24F IPD cases from 1999 – 2016 used for calculating the serotype 24F incidence data.

| Year | Number of people 0 - 4 year | 0 - 4 year with IPD | Incidence | Year | Number of people 5 - 64 year | 5 - 64 year with IPD | Incidence |
| --- | --- | --- | --- | --- | --- | --- | --- |
| 1999 | 344685 | 2 | 0.580239929 | 1999 | 4178425 | 3 | 0.071797 |
| 2000 | 340593 | 0 | 0 | 2000 | 4199025 | 4 | 0.09526 |
| 2001 | 337589 | 1 | 0.296218182 | 2001 | 4219795 | 5 | 0.118489 |
| 2002 | 335507 | 2 | 0.596112749 | 2002 | 4238263 | 7 | 0.165162 |
| 2003 | 332056 | 2 | 0.602308044 | 2003 | 4253100 | 5 | 0.117561 |
| 2004 | 330377 | 1 | 0.302684509 | 2004 | 4262685 | 1 | 0.023459 |
| 2005 | 328056 | 1 | 0.304826005 | 2005 | 4270846 | 6 | 0.140487 |
| 2006 | 325152 | 0 | 0 | 2006 | 4279280 | 1 | 0.023368 |
| 2007 | 324883 | 2 | 0.615606234 | 2007 | 4287456 | 5 | 0.116619 |
| 2008 | 325606 | 1 | 0.307119648 | 2008 | 4297144 | 2 | 0.046543 |
| 2009 | 326932 | 1 | 0.305874004 | 2009 | 4309023 | 2 | 0.046414 |
| 2010 | 326067 | 2 | 0.613370872 | 2010 | 4305812 | 3 | 0.069673 |
| 2011 | 325495 | 0 | 0 | 2011 | 4301352 | 5 | 0.116243 |
| 2012 | 319203 | 2 | 0.626560527 | 2012 | 4293229 | 10 | 0.232925 |
| 2013 | 312956 | 6 | 1.917202418 | 2013 | 4289871 | 8 | 0.186486 |
| 2014 | 303782 | 11 | 3.621017703 | 2014 | 4296719 | 4 | 0.093094 |
| 2015 | 298368 | 5 | 1.675782926 | 2015 | 4310218 | 20 | 0.464014 |
| 2016 | 294748 | 4 | 1.357091482 | 2016 | 4338081 | 6 | 0.13831 |
|  |  |  |  |  |  |  |  |
| Year | Number of people 65+ year | 65+ with IPD | Incidence | Year | Number of people all age group | Total with IPD | Total incidence |
| 1999 | 790467 | 9 | 1.138567454 | 1999 | 5313577 | 14 | 0.263476 |
| 2000 | 790402 | 4 | 0.506071594 | 2000 | 5330020 | 8 | 0.150093 |
| 2001 | 791828 | 6 | 0.757740317 | 2001 | 5349212 | 12 | 0.224332 |
| 2002 | 794584 | 4 | 0.503408073 | 2002 | 5368354 | 13 | 0.24216 |
| 2003 | 798351 | 9 | 1.127323696 | 2003 | 5383507 | 16 | 0.297204 |
| 2004 | 804578 | 7 | 0.870021303 | 2004 | 5397640 | 9 | 0.16674 |
| 2005 | 812503 | 5 | 0.615382343 | 2005 | 5411405 | 12 | 0.221754 |
| 2006 | 823027 | 5 | 0.607513484 | 2006 | 5427459 | 6 | 0.110549 |
| 2007 | 834745 | 8 | 0.95837651 | 2007 | 5447084 | 15 | 0.275377 |
| 2008 | 853041 | 5 | 0.586138298 | 2008 | 5475791 | 8 | 0.146098 |
| 2009 | 875496 | 9 | 1.027988706 | 2009 | 5511451 | 12 | 0.217729 |
| 2010 | 902859 | 7 | 0.775314861 | 2010 | 5534738 | 12 | 0.216812 |
| 2011 | 933781 | 6 | 0.642548949 | 2011 | 5560628 | 11 | 0.197819 |
| 2012 | 968084 | 20 | 2.065936427 | 2012 | 5580516 | 32 | 0.573424 |
| 2013 | 999801 | 8 | 0.800159232 | 2013 | 5602628 | 22 | 0.392673 |
| 2014 | 1026734 | 27 | 2.629697663 | 2014 | 5627235 | 42 | 0.74637 |
| 2015 | 1051129 | 8 | 0.761086413 | 2015 | 5659715 | 33 | 0.583068 |
| 2016 | 1074422 | 15 | 1.396099484 | 2016 | 5707251 | 25 | 0.438039 |
